# Supplementary material for: Predicting IVF outcomes using a logistic regression–ABC hybrid model: A proof-of-concept study on supplement associations
Source: PLoS One. 2025 Nov 25;20(11):e0336846. doi: 10.1371/journal.pone.0336846 (PMC12646469; doi:10.1371/journal.pone.0336846)
Supplement: S1 Appendix — (DOCX) [file pone.0336846.s001.docx]

**Table A1.** The Nutritional and pharmaceutical supplements that fully meet daily needs

| The following list appeared in the survey | Active ingredient | An active ingredient that meets your daily needs at a rate of 100%. |
| --- | --- | --- |
| Astalife | Astalife Fish Oil, DHA, Astaxanthin, Fish Oil | DHA, omega-3 |
| Folic acid | Folic acid | Folic acid |
| DHA | DHEA | DHA |
| Q10 | Coenzyme Q10 | Coenzyme Q10 |
| Omega-3 | Omega-3 | Omega-3 |
| DHEA | DHEA | DHA |
| EPA |  | EPA |
| DHEA | DHEA | DHEA |
| Sidefer | Vitamin B12, ferritin, Vitamin C, folic acid, Vitamin B5 | Vitamin B12, ferritin, Vitamin C, folic acid, Vitamin B5 |
| Elenatal | Multi Vitamin, mineral and folic acid Metafolin | Vitamin B12, ferritin, Vitamin C, folic acid, Vitamin B6 |
| Ferzsenol | Ferritin III hidroksit, polimaltoz complex | Ferritin |
| D vit | D Vitamin | Vitamin D |
| Resveratrol | A natural phytoalexin | Phytoalexin |
| Magnesium | Magnesium | Magnesium |
| Biosteron | DHEA | DHA |
| Proceive f | inositol, coenzyme Q10, glutatyon | Vitamin B12, ferritin, Vitamin C, folic acid, Vitamin B6,Vitamin D |
| Ferritin d3 | Ferritin III hidroksit | Ferritin |
| Biosleron | Dhea | DHA |
| Diofol | Folic acid, metafolin, B12, D3 Vitamins and iyot | Folic acid, metafolin, Vitamin B12, Vitamin D3 |
| Selenium | Selenium | Selenium |
| Argufertil | Coenzyme Q10, Folic acid, B12, C Vitamin, Zinc, Selenium | Coenzyme Q10, folic acid, Vitamin B12, zinc, selenium |

**Table A2.** Detailed comparison of baseline and hybrid machine learning models in predicting IVF outcomes (%).

| **No** | **Stage** | **Model** | **Acc.** | **Std** | **F-Score** | **Std** | **Recall** | **Std** | **Precision** | **Std** | **Model Type** |
| --- | --- | --- | --- | --- | --- | --- | --- | --- | --- | --- | --- |
| 1 | 1 | Random Forest | 85.19 | 7.16 | 84.79 | 7.30 | 85.95 | 7.46 | 84.74 | 6.86 | Simple |
| 2 | 1 | SVM | 84.55 | 4.43 | 83.80 | 4.38 | 83.84 | 4.06 | 84.00 | 4.68 | Simple |
| 3 | 1 | CART | 81.44 | 4.53 | 80.70 | 4.84 | 81.28 | 4.99 | 80.68 | 4.84 | Simple |
| 4 | 1 | KNN | 63.56 | 7.31 | 61.38 | 8.59 | 62.43 | 8.61 | 62.54 | 9.26 | Simple |
| 5 | 2 | LR - RF | 89.49 | 4.68 | 89.17 | 4.70 | 90.19 | 4.86 | 89.18 | 4.57 | Hybrid |
| 6 | 2 | LR - KNN | 86.40 | 5.47 | 85.87 | 5.64 | 86.55 | 6.06 | 85.76 | 5.40 | Hybrid |
| 7 | 2 | LR - SVM | 82.67 | 5.19 | 81.34 | 6.21 | 81.34 | 6.53 | 82.45 | 5.32 | Hybrid |
| 8 | 2 | LR - CART | 85.80 | 5.41 | 84.81 | 6.00 | 84.81 | 6.38 | 85.71 | 5.13 | Hybrid |
| 9 | 3 | ABC-LR-RF | **91.36** | 5.57 | **90.57** | 5.62 | **96.92** | 4.48 | **85.62** | 4.23 | Hybrid |
| 10 | 3 | ABC-LR-KNN | 87.67 | 5.08 | 85.07 | 5.21 | 88.97 | 5.11 | 89.34 | 5.35 | Hybrid |
| 11 | 3 | ABC-LR-CART | 90.13 | 5.15 | 90.20 | 5.13 | 95.38 | 6.31 | 87.11 | 4.08 | Hybrid |
| 12 | 3 | ABC-LR-SVM | 88.26 | 5.17 | 84.55 | 5.21 | 89.10 | 4.87 | 85.63 | 3.77 | Hybrid |

**Table A3.** Determination of the hyperparameters used in ABC algorithms (%)**.**

| Fitness Function | Bee Count | Iteration | Acc. | Bee Count | Iteration | Accuracy |
| --- | --- | --- | --- | --- | --- | --- |
| Random Forest | 5 | 10 | 88.30 | 5 | 20 | 84.37 |
|  | 10 | 10 | 88.30 | 10 | 20 | 88.30 |
|  | 15 | 10 | 89.51 | 15 | 20 | 90.13 |
|  | 20 | 10 | 88.30 | 20 | 20 | 86.19 |
|  | 5 | 30 | 85.63 | 5 | 40 | 85.50 |
|  | 10 | 30 | 88.30 | 10 | 40 | 88.30 |
|  | **15** | **30** | **91.36** | 15 | 40 | 90.13 |
|  | 20 | 30 | 85.63 | 20 | 40 | 86.19 |

| **Algorithm 1:** ABC LR Hybritization | | | | | | | |  |  |
| --- | --- | --- | --- | --- | --- | --- | --- | --- | --- |
|  |  | **Set** X | | | | // Full dataset | | |  |
|  |  | **Set** y | | | | // Binary class labels | | |  |
|  |  | **Set** num_bees | | | | // Number of bees in the colony | | |  |
|  |  | **Set** max_iter | | | | // Maximum number of ABC iterations | | |  |
|  |  | **Set** abandonment_limit | | | | // Limit after which scout bees are triggered | | |  |
|  |  | **Set** k_folds | | | | // Number of CV folds (5 fold) | | |  |
|  |  |  | | | |  | | |  |
| **1.** |  | **BEGIN:** | | | |  | | |  |
| **2.** |  | - **Initial Feature Selection using Logistic Regression** | | | | | | |  |
| **3.** |  | - **Select features with significant coefficients** | | | | | | |  |
| **4.** |  | - **Define X_reduced ← X with selected features only** | | | | | | |  |
| **5.** |  |  | | | | |  | |  |
| **6.** |  | **Initialize Stratified K-Fold CV:** | | | | |  | |  |
| **7.** |  | - **Split X_reduced and y into k_folds maintaining class proportions** | | | | | | |  |
| **8.** |  |  | | | | | | |  |
| **9.** |  | **Optimization Loop (ABC Algorithm):** | | | | | | |  |
| **10.** |  | **Initialize population** | | | | | | |  |
| **11.** |  | **For** each bee **in** num_bees**:** | | | | | | |  |
| **12.** |  |  | Generate random binary vector | | | (length = number of features in X_reduced) | | | |
| **13.** |  |  | Evaluate fitness_i as follows: | | | | | | |
| **14.** |  |  | **For** each train_index, val_index **in** StratifiedKFold: | | | | | | |
| **15.** |  |  |  | | **Split** X_train, X_val and y_train, y_val | | | | |
| **16.** |  |  |  | | **Apply** SMOTE on (X_train, y_train) to balance classes | | | | |
| **17.** |  |  |  | | Select features **where** binary vector = 1 🡺 X_train_selected, X_val_selected | | | | |
| **18.** |  |  |  | | **Fit** the model on a balanced training set | | | | |
| **19.** |  |  |  | | **Compute** validation performance (accuracy, F1-score, recall, precision) | | | | |
| **20.** |  |  | **Average** the metric across folds 🡺 fitness_i | | | | | | |
| **21.** |  |  |  | | | | | | |
| **22.** |  |  | **Repeat** for **max_iter** | | | | | | |
| **23.** |  |  |  | | Employed Bee Phase 🡺 Explore neighbors and update if better | | | | |
| **24.** |  |  |  | | Onlooker Bee Phase 🡺 Probabilistically improve good solutions | | | | |
| **28.** |  |  |  | | Scout Bee Phase 🡺 Replace stagnated solutions | | | | |
| **27.** |  |  |  | | | | | | |
| **28.** |  |  | **After ABC convergence:** | | | | | | |
| **29.** |  |  | Select best-performing bee solution 🡺 best_features | | | | | | |
| **30.** |  |  |  | |  | | | | |
| **31.** |  | **RETURN** | | | | | | | |
| **32.** |  |  | | best_features | | | | |  |
| **33.** |  | **END** | | | | | | |  |

**Equations of the ABC algorithms**

Minimize or Maximize $f\left( x \right)$subject to x ∈ $Ɍ^{D}$. In our model, we maximize the $f\left( x \right)$

where

$x={[\boldsymbol{x}_{\boldsymbol{1}}\boldsymbol{,}\boldsymbol{x}_{\boldsymbol{2}}\boldsymbol{,\ldots,}\boldsymbol{x}_{\boldsymbol{D}}]}$ , $f\left( x \right)$ is the fitness function, D is the number of predictor

**Population Initialization Phase**

Each solution is represented by a vector

$x={[x_{1i}, x_{i2},\ldots, x_{iD}]}$ i= 1,2,…, N (1)

$$\boldsymbol{x}_{\boldsymbol{ij}}\boldsymbol{=}x_{j}^{min}\boldsymbol{+}rand\left( 0,1 \right)\boldsymbol{.(}x_{j}^{max}\boldsymbol{-}x_{j}^{min}\boldsymbol{)}$$

where

N is number of employed bees/Number of solution(food)

**Employed Bee Phase**

Each employed bee generates a new solution $\boldsymbol{v}_{\boldsymbol{ij}}$using

${v_{ij}= x_{ij}+ ⌽_{ij} .(x_{ij}-x_{kj})}$ (2)

where

- k ∈ $\left\{ 1,2,\ldots.,N \right\}, k\neq i$
- j ∈ $\left\{ 1,2,\ldots., D \right\} randomly chosen dimension$
- $\boldsymbol{⌽}_{\boldsymbol{ij}}$ ∈ [-1,1] *is a random number*

Apply selection ;

${\boldsymbol{x}_{\boldsymbol{i}}}=\left\{ \begin{aligned} \boldsymbol{x}_{\boldsymbol{i}}, &\mathrm{if}f\left( v_{\boldsymbol{i}} \right) is better than f\left( x_{\boldsymbol{i}} \right) \\ v_{\boldsymbol{i}}, &otherwise \end{aligned} \right.$ (3)

**Onlooker Bee Phase**

Onlooker bees select a solution $x_{i}$ , probability of selection

$p_{i}=\frac{{f_{i}t_{i}}}{\sum_{k=1}^{N} {{f_{i}t_{k}}}}$ (4)

**Scout Bee Phase**

In the event that a solution does not improve after a specified number of trials (limit), it is abandoned and replaced.

$\boldsymbol{x}_{\boldsymbol{ij}}\boldsymbol{=}x_{j}^{min}\boldsymbol{+}rand\left( 0,1 \right).(x_{j}^{max}\boldsymbol{-}x_{j}^{min}\boldsymbol{)}$

**Fitness Calculation**

Objective function $f\left( x \right)$ is transformed to fitness:

$$fit\left( x \right)=\left\{ \begin{aligned} \frac{1}{1+f\left( x \right)}, &\mathrm{if}f\left( \boldsymbol{x} \right)\geq0 \\ 1+|f\left( x \right)|, \mathrm{if}f\left( \boldsymbol{x} \right)<0 \end{aligned} \right.$$

The algorithm stops when a maximum number of iterations is reached or a convergence threshold is satisfied.
