# Supplementary material for: Predicting IVF outcomes using a logistic regression–ABC hybrid model: A proof-of-concept study on supplement associations
Source: PLoS One. 2025 Nov 25;20(11):e0336846. doi: 10.1371/journal.pone.0336846 (PMC12646469; doi:10.1371/journal.pone.0336846)
Supplement: Attachment — Submitted filename: Responds to reviewer_2.docx [file pone.0336846.s004.docx]

**Responds to reviewer**

Reiewer 2 : 1- Clarify dietary data inclusion (or justify exclusion).

Response : We thank the reviewer for this important observation. In the original submission, only supplement intake was encoded as binary “active ingredient” variables, while detailed dietary intake data were not included. The exclusion of dietary data was due to the absence of reliable and standardized dietary intake records in the available patient dataset. This acknowledgement has been incorporated into the revised manuscript, specifically within the section addressing research limitations.

Reiewer 2 : 2-	Temper conclusions about hybrid model superiority.

Response : We thank the reviewer for this helpful suggestion. In the revised version of the manuscript, the conclusion section has been redesigned and is now highlighted in colour.

Reiewer 3 : 1- The sample size (N=162, with only 33 used for testing initially) is critically small for a machine learning study with 21+ input variables. This is especially true for a hybrid model involving a metaheuristic optimizer (ABC), which is prone to overfitting. While the use of 5-fold cross-validation mitigates this to some degree, the absolute number of samples remains a severe limitation. The results, particularly the very high accuracy and F-scores (~90%), are highly suspect and likely reflect overfitting to the specific cohort rather than a generalizable model. The generalizability of the findings is extremely limited. Claims of model efficacy (e.g., 91% accuracy) are not credible for real-world clinical application based on this dataset alone.

Response : We thank the reviewer for highlighting this important concern. We agree that the relatively small sample size (N = 162, with only 33 test cases) poses a severe limitation and increases the risk of overfitting, particularly for hybrid ABC models. To address this, we have added a dedicated Research Limitations section where we explicitly acknowledge the limited cohort size, the risk of inflated accuracy, and the need for validation on larger, multi-center datasets before clinical application. We also emphasize that our findings should be interpreted as exploratory and methodological, rather than as generalizable clinical recommendations.

Reiewer 3 : 2- The process of transforming drug names into "active ingredients" is the study's most novel aspect, but remains its biggest weakness. The method described (labeling a patient as "1" for a supplement if their intake meets 100% of the daily requirement) is overly simplistic and clinically naive. This binary transformation completely ignores the dosage (was it 100% or 500% of the daily requirement?) and duration (taken for a week vs. a year) of supplementation, which are fundamental to its biological effect. This renders the "active ingredient" variables nearly meaningless from a clinical perspective.
As pointed out by a reviewer, the patient's baseline dietary intake of these nutrients is not taken into account. A patient eating a diet rich in Omega-3s might be labeled "0" for not taking a supplement, while their actual nutrient levels could be high. The identified "key factors" (Omega-3, Folic Acid) are likely correct based on established literature, but the study's methodology does not provide robust, novel evidence to support this. It merely shows that a crude binary representation of supplement use has predictive value in a small, overfit model.

Response : We thank the reviewer for highlighting this important methodological limitation. We fully agree that the binary transformation of supplements into “active ingredient” variables is clinically simplistic, as it does not capture dosage, duration of use, or baseline dietary intake. In the revised manuscript, we have reinforced the Research Limitations section by highlighting it in colour to emphasise that this approach makes the supplementary variables clinically imprecise and potentially inaccurate. We now explicitly note that, although our model identified factors such as omega-3 and folic acid consistent with the literature, these results should be regarded as exploratory, hypothesis-generating correlations rather than robust clinical evidence. We also highlight the need for future studies to incorporate quantitative dosage, duration, and baseline nutrition data in order to produce clinically meaningful predictors.

Reiewer 3 : 3- The performance metrics reported are difficult to trust due to the high risk of overfitting. The improvement from simple models (e.g., RF: 85.19%) to hybrid models (e.g., ABC-LR-RF: 90.73%) is marginal and could easily be due to random chance, especially given the small sample size and the use of cross-validation within the optimization loop. An ablation study showing the standalone contribution of the ABC algorithm is missing. The recall for ABC-LR-RF is reported as 95.38%, which is astronomically high for a biological outcome like IVF success and is a classic red flag for overfitting or data leakage. The central claim of the paper—that the hybrid ABC model is highly effective—is not sufficiently proven. The results section reads more like an optimization exercise than a robust validation of a predictive model.

Response : We thank the reviewer for raising these crucial concerns regarding model performance and potential overfitting. We agree that the small cohort size and the marginal improvements observed between the simple and hybrid models limit the robustness of our conclusions. In the revised manuscript, we have clarified in both the 'Results' and 'Research Limitations' sections, using highlighted colour, that the observed improvements in accuracy and recall may be partially attributable to chance and cannot be interpreted as definitive evidence of superior model performance. We have also added explicit caution that the unusually high recall values, while numerically obtained under stratified cross-validation with SMOTE, are likely inflated and should be regarded as a red flag for potential overfitting rather than as clinically valid outcomes.

Regarding the absence of an ablation study, we acknowledge this as a limitation and now explicitly state that future work should include standalone evaluations of the ABC algorithm’s contribution to validate its incremental value beyond traditional models. We have revised our conclusions to present the hybrid ABC approach not as a validated clinical model, but rather as an exploratory optimization framework whose methodological novelty requires further confirmation in larger, multi-center datasets.

Reiewer 3 : 4- While the authors acknowledge this in the discussion (a good addition from the revision), the entire framing of the paper risks implying causation. The title says ‘to treat subfertility,’ and the conclusion identifies ‘the most significant supplements.’ However, the model is built on observed supplement use correlated with success. This could easily be reversed: clinicians may be more likely to prescribe these supplements to patients with a better prognosis, or more affluent/health-conscious patients (who have better outcomes) are more likely to take them. The model cannot disentangle this. The clinical recommendations are overstated. The study identifies associations, not treatment effects.

Response : We thank the reviewer for this important clarification regarding causality. We agree that our study is observational and retrospective, and therefore cannot make causal inferences or clinical recommendations. In response, we have revised the framing throughout the manuscript:

- The title has been changed to emphasize the exploratory, methodological nature of the work rather than suggesting treatment effects (page 1).
- A highlighted color has been added to the end of the conclusion section to emphasize that the study finds correlations and associations rather than causal treatment effects.
- We now explicitly note that the observed associations may reflect underlying confounding factors such as clinician prescribing patterns or patient socioeconomic/health profiles, and that the model cannot disentangle these effects

Therefore, before any clinical conclusions can be made, our findings should only be presented as exploratory, hypothesis-generating associations that need to be confirmed in prospective or randomized studies.

Reiewer 3 : 5- While the authors have now shared code and data (a major improvement), the quality of the documentation is poor. The GitHub repository contains a Jupyter notebook (Gebelik_calisma_revision.ipynb) but no README.md file explaining how to run it, the required dependencies, or the structure of the data. The manuscript itself is riddled with minor errors (e.g., ‘Random Forrest,’ ‘The Informations,’ ‘Folic acid 3’ in Table 2, inconsistent numbering from Table 1 to Table 2), which reduce confidence in the overall rigor. Although technically ‘available,’ the work is difficult to reproduce or build upon.

Response : We thank the reviewer for recognizing our effort to make both the data and code publicly available, and we fully agree that proper documentation and presentation are essential for reproducibility and clarity. In response to this valuable feedback, we have made the following improvements:

- Added a README.md file in the GitHub repository that clearly explains how to run the Jupyter notebook, the required dependencies (via a requirements.txt file), and the structure of the dataset.
- Uploaded a requirements.txt file so that all dependencies can be installed easily, ensuring full reproducibility of the analysis.
- Carefully proofread and corrected minor errors in the manuscript, including replacing “Random Forrest” with “Random Forest,” “The Informations” with “The Information,” correcting “Folic acid 3” to “Folic acid” in Table 2, and fixing inconsistent numbering between Table 1 and Table 2.
- Revised the Methods section to provide additional clarification of the dataset structure and preprocessing steps, consistent with the documentation in the GitHub repository.

We believe these changes substantially improve the rigor, clarity, and reproducibility of the work.

Reiewer 3 : 6- Figure Quality: The figures (as described in the text) are still problematic. For example, Figure 1 is described as a ‘flow diagram of the conversion of drugs into active substances’ but its caption (on Page 38) is garbled (‘Import loop... Local power to add follow-up devices’). This suggests the figures were not properly finalized.

Response: We thank the reviewer for pointing out this error. We upload again the quality form of the each figures. But maybe when the system converted to pdf for review, System broke the resolution of the Figures. We sincerely apologize for the oversight. In the revised manuscript, we have carefully reviewed and corrected all figure captions to ensure accuracy and consistency with the text. Specifically, the caption for Figure 1 has been corrected to:

*“Figure 1. Flow diagram of the conversion of drugs into active substances.”*

We have also double-checked all other figures (Figures 2–6) to ensure that their captions and numbering are consistent, descriptive, and finalized.

Reiewer 3 : 7- ABC Parameter Justification: The choice of ABC parameters (20 bees, 10 iterations) is arbitrary and not justified. A sensitivity analysis would be needed to show these are appropriate.

Response: We thank the reviewer bringing up this crucial methodological issue. We admit that the original version did not adequately explain the choice of ABC parameters (20 bees, 10 iterations). We now offer a rationale in the updated manuscript that is supported by both previous research and computational viability. In particular, comparable swarm sizes (20–50 bees) and iteration ranges (10–50) have been documented as effective defaults in prior ABC applications in biomedical and optimization contexts (e.g., [Karaboga & Basturk, 2007]; [Akay & Karaboga, 2012]). We chose modest values (20 bees, 10 iterations) to minimize computational cost while guaranteeing convergence, considering the exploratory nature of our study and the relatively small dataset (N = 162). We chose modest values (20 bees, 10 iterations) to minimize computational cost while guaranteeing convergence, considering the exploratory nature of our study and the relatively small dataset (N = 162). We acknowledge that a sensitivity analysis would improve the study, but this version did not have the capacity to conduct one because of dataset size limitations. We have made it clear that this is a limitation and that, in order to confirm their robustness, future studies should examine the effects of changing ABC parameters in a methodical manner.

Reiewer 3 : 8- Result Presentation: Table 3 is busy and difficult to understand. A summary table showing the best model for each algorithm type would be clearer.

Response: We thank the reviewer for this helpful suggestion. We agree that the original Table 3 was overly detailed. In the revised manuscript, we have addressed this in two ways:

1- For the sake of transparency, we have marked the important parts of Table 3 in bold.
2- Furthermore, we developed a new summary table (Table 4) that solely displays the top-performing model for every kind of algorithm (e.g., baseline vs. LR-hybrid vs. ABC-hybrid). The full table is available for those who want to look at every detail, but this summary offers a concise, easy-to-read summary of the key findings.

Reiewer 3 : 9- The title and conclusions must be tempered. Instead of ‘identifying effective ingredients to treat,’ frame it as ‘identifying associations between supplement use and IVF outcomes using a novel hybrid ML approach.’ Emphasize the methodological contribution over the clinical recommendations

Response : We thank the reviewer for this constructive feedback. We agree that the previous version of our manuscript overstated the clinical implications. Following your suggestion, both the title and the conclusion have been revised to emphasize the methodological contribution and to frame the findings as exploratory associations rather than treatment recommendations.

Original Title:

“Identifying Effective Ingredients to Treat Subfertility with a Hybrid Machine Learning–Metaheuristic Model”

Revised Title:

“Predicting IVF Outcomes Using a Logistic Regression–ABC Hybrid Model: A Proof-of-Concept Study on Supplement Associations”

Original Conclusion (excerpt):

“Omega 3 and folic acid have been identified as the most important dietary supplements in the treatment of subfertility, and the proposed model provides strong evidence for their clinical application.”

Revised Conclusion (excerpt):

“In conclusion, even though the model identified folic acid and omega-3 as predictive variables, these findings should be regarded as exploratory correlations rather than specific treatment suggestions. This work's main innovation is its methodological framework, a hybrid Logistic Regression–Artificial Bee Colony (LR-ABC) model that combines feature selection and metaheuristic optimization to investigate the results of IVF. Therefore, this study should be considered a proof-of-concept demonstration of the potential of ML–ABC approaches in reproductive medicine. Before making clinical judgments, validation with larger, multi-center datasets that include dosage, duration, and baseline dietary intake will be required in the future.”

Reiewer 3 : 10- Acknowledge the severe limitation of the binary supplement variable. Discuss this as a major limitation of the current study and propose how future work with more detailed data (dose, duration, baseline diet) could overcome it.

Response : We have now explicitly acknowledged this as a major limitation in the Conclusion and Research Limitations sections. In addition, we have proposed that future studies should collect more detailed data on supplement dosage, duration of use, and baseline dietary intake, ideally through prospective or randomized designs. Incorporating these factors will allow for more accurate modeling of nutrient effects and strengthen the biological and clinical validity of the approach.

Reiewer 3 : 11- Remove the emphasis on the 91% accuracy claim. Instead, focus on the comparative performance between models and the utility of the LIME explanations for generating hypotheses.

Response: Thank you for your valuable contribution. In the revised abstract, results and discussion, and conclusion, which were highlighted in the revised manuscript in colour, the emphasis on the 91% accuracy figure has been removed. Instead, the focus has been shifted to the comparative performance of hybrid ABC-based models relative to baseline models, and to the utility of LIME explanations for generating interpretable, hypothesis-driven insights. The conclusion now positions the study as predominantly a methodological contribution rather than asserting clinical efficacy.

Reiewer 3 : 12- Clean up the code repository. Add a detailed README.md file, ensure the code is well-commented, and verify that the provided data file matches the one used to generate the results in the paper

Response: We thank the reviewer for this valuable suggestion. In the revised submission, we have thoroughly cleaned and reorganized the code repository in github.We created a detailed README file that documents the repository structure, data schema, preprocessing steps, model training commands, LIME explanation generation, and test suite instructions. Ww exported the stratified 5-fold cross-validation indices used in the study and included them in the repository. We also provided scripts for generating manuscript figures, with sample data.

Reiewer 3 : 13- A thorough proofread by a native English speaker is essential to fix grammatical errors, typos, and inconsistent terminology throughout the manuscript.

Response: Thank you for this valuable suggestion. We have carefully revised the manuscript to eliminate repetitions and correct grammatical errors. In addition, the paper has undergone thorough proofreading to ensure that it meets academic writing standards in terms of clarity, style, and consistency. These improvements enhance the overall readability and professionalism of the manuscript.
