# Supplementary material for: Predicting IVF outcomes using a logistic regression–ABC hybrid model: A proof-of-concept study on supplement associations
Source: PLoS One. 2025 Nov 25;20(11):e0336846. doi: 10.1371/journal.pone.0336846 (PMC12646469; doi:10.1371/journal.pone.0336846)
Supplement: Attachment — Submitted filename: Responds to reviewer_3.docx [file pone.0336846.s005.docx]

**Responds to reviewer**

Reviewer 2 : 1- Make the limitations section stronger to indicate more clearly that the binary supplement coding cannot reveal the real nutrient status or biological outcome.

Response : We thank the reviewer for this important observation. We enlarge and detailed the Research limitation section

“ *Our exposure variables for micronutrient use were encoded binarily (e.g., supplement taken vs. not taken at a threshold such as ≥100% RDI). This encoding is pragmatic but cannot recover true nutrient status or biological effect for several reasons. First, it discards dose and duration information, precluding any dose–response assessment and ignoring cumulative exposure. Second, it assumes a uniform effect across brands and formulations, while bioavailability varies markedly with chemical form, excipients, co-ingested foods, and timing of intake. Third, binary use does not reflect adherence (frequency/consistency) or timing relative to the biological window of interest (e.g., peri-procedural vs. long-term use), both of which influence physiological impact. Fourth, we lack baseline nutritional status and objective biomarkers (e.g., serum folate, DHA, vitamin D); thus, we cannot distinguish deficiency correction from supraphysiologic exposure, nor can we account for inter-individual differences in absorption and metabolism. Finally, supplement use is correlated with broader health behaviors and socioeconomic factors; with only binary indicators, residual confounding and non-differential misclassification are likely, which can attenuate or unpredictably bias associations.* ”

.

Reviewer 2 : 2- Include some commentary on possible confounding variables (socioeconomic status, access to healthcare, clinician prescribing patterns) that might be the cause of the observed associations.

Response : We thank the reviewer for this helpful suggestion. In the revised version of the manuscript, the Research Limitations section has been redesigned and is now highlighted in colour.

*“ Our observed associations may be partly explained by unmeasured confounding. First, socioeconomic status (SES)—including education, income, insurance coverage, and neighborhood deprivation—can influence both supplement use (ability to purchase higher-quality products, better adherence) and clinical outcomes (health literacy, earlier presentation, healthier baseline). Second, access to healthcare (clinic proximity, appointment availability, out-of-pocket costs, private vs. public care) may differentially shape care pathways and follow-up, thereby confounding the link between supplement use and outcomes. Third, clinician prescribing patterns introduce confounding by indication: clinicians may recommend or escalate supplementation preferentially for patients they judge at higher (or lower) risk based on unrecorded clinical cues; practice style, brand/formulation preferences, and evolving guidelines can also vary across clinicians and time. Together with other lifestyle and clinical factors that we could not fully measure (diet quality, physical activity, smoking, comorbidities, baseline nutrient status, time-to-treatment, lab protocols, and calendar-time effects), these sources of confounding and non-differential misclassification of exposure may attenuate or bias associations in unpredictable directions. Accordingly, findings should be interpreted as associations with reported supplement use rather than causal effects. “*

Reviewer 2 : 3- Once more, update the abstract and conclusion to make it clear that this is a methodological demonstration, but not clinically practical results.

Response :

*“ The objective of this study is to investigate predictive signals for outcomes in IVF/ART. The study presents a method-focused workflow that combines engineered clinical variables with an ABC-assisted selection/tuning strategy. The findings suggest that in a controlled, leakage-safe assessment, regularly recorded variables, such as binary supplement indicators, can facilitate associational modelling. Importantly, clinical use is not the goal of these models. The lack of dose, duration, adherence, dietary intake, and objective biomarkers restricts interpretability, and binary coding of supplementation cannot recover true nutrient status or biological effect. Furthermore, some of the observed associations may be explained by unmeasured confounding, such as socioeconomic status, access to healthcare, clinician prescribing patterns and indications, and lifestyle factors. The current findings should be interpreted as proof-of-concept rather than practical advice due to these limitations and the size of the dataset. “*

Reviewer 3 : 1- The sample size of N=162 (with 33 test cases) is critically small for ML with 21+ predictors. The reported performance (accuracy ~91%, recall ~95%) is likely inflated due to overfitting, even with SMOTE and cross-validation. Lack of external validation or an independent test dataset limits the credibility of generalization.

Respond : Thank you for your valuable comment. The process of data collection for this study is both protracted and laborious. The collection of this data was made possible by the utilisation of limited resources available in the designated small area.

We added explanation to the Research limitations and future works section.

*“ Notwithstanding these methodological precautions, the limited sample size indicates that the accuracy and F-scores reported should be interpreted with caution and are unlikely to be applicable to broader clinical populations without further validation. The modest dataset size restricts the generalisability of the findings, and the reported performance metrics should be interpreted with caution”*

## Reviewer 3 : 2- Binary transformation of supplements into “active ingredient = 1 if ≥100% daily requirement” is clinically simplistic and potentially misleading. The paper ignores dosage, duration, and baseline diet/nutritional status, which undermines biological validity.

Respond : Thank you for your valuable suggestion. We revised the Research limitations and future works section. The following paragraph constitutes our response to the proposal

Thirdly, only supplement intake was encoded as binary “active ingredient” variables (coded as 1 if reported intake met 100% of the recommended daily intake). Detailed dietary intake data (e.g., habitual nutrient consumption from food sources) were not included. This exclusion was due to the unavailability of reliable, standardized dietary records in the patient dataset. The conversion of drug and supplement intake into "active ingredient" variables was streamlined into a binary coding system, designating patients as "1" if their reported intake satisfied all daily requirements. Notably, this method overlooks crucial clinical information, including dosage levels (for instance, 100% versus 500% of the recommended intake) and usage duration (for instance, short-term versus long-term supplementation). Furthermore, the absence of baseline dietary intake data is a notable limitation. To illustrate, a patient who naturally consumes a diet high in omega-3 fatty acids but does not take supplements would be assigned the label "0," despite their actual nutrient levels being adequate. These constraints serve to reduce the biological precision of the "active ingredient" variables This simplification renders the supplement variables clinically simplistic and potentially misleading, as it ignores fundamental determinants of biological effect such as dose, duration, and baseline nutrition. Therefore, while the identification of omega-3 and folic acid as predictive factors is consistent with established literature, these findings should be interpreted as exploratory, hypothesis-generating correlations rather than as robust novel clinical evidence.

Reviewer 3 : 3- No ablation study was performed to isolate the contribution of the ABC optimizer vs. LR baseline. Reported marginal improvements (e.g., RF 85% → 90.7%) may be due to chance. Despite revisions, some language still risks implying causality (e.g., describing omega-3 as “effective” for subfertility). Confounding factors (prescriber bias, socioeconomic status, and underlying prognosis) are not adequately accounted for.

Respond : Thank you your valuable comment. We sincerely thank the reviewer for these insightful comments regarding both methodological clarification and interpretive precision.

1. **Ablation analysis:**

We acknowledge that an ablation or sensitivity analysis to isolate the specific contribution of the Artificial Bee Colony (ABC) optimizer relative to the Logistic Regression (LR) baseline was not performed. In response, we have explicitly addressed this limitation in the revised *Discussion* . The text now clarifies that the modest performance improvements observed (e.g., RF 85.2% → 90.7%) may partly reflect stochastic variation rather than a definitive optimization advantage. The revised manuscript also notes that future work should include a controlled ablation design to evaluate the independent effect of the ABC component under identical resampling conditions.

2. **Causality and confounding:**

We carefully reviewed all phrasing that might imply causal inference and revised the manuscript accordingly. Expressions such as “omega-3 is effective” have been replaced with non-causal, association-oriented phrasing (e.g., “omega-3 use was identified as a predictive variable”). Furthermore, we have added a new paragraph in the *Discussion* emphasizing that supplement-related associations may be influenced by confounding factors such as prescriber bias, socioeconomic status, healthcare access, and underlying prognosis. The revised text explicitly states that the findings are exploratory and hypothesis-generating rather than indicative of therapeutic efficacy.

We believe these changes directly and comprehensively address the reviewer’s concerns, ensuring that the study’s methodological and interpretive scope is presented with appropriate caution and clarity.

Below explanationwas added to discussion section.

*“ Despite the fact that the hybrid models attained comparatively higher levels of predictive performance, the absence of an ablation or sensitivity analysis hinders the capacity to ascertain the independent contribution of the Artificial Bee Colony (ABC) optimizer with respect to the Logistic Regression (LR) baseline. This is evidenced by the observed performance improvements (e.g., an enhancement in Random Forest accuracy from 85.2% to 90.7%) that may be attributable to stochastic variation rather than a distinct optimization advantage. Moreover, it is imperative to refrain from interpreting the associations identified in this study as causal. A number of variables have been found to be predictive of outcomes, including omega-3 use, folic acid intake, and dietician support. However, these effects may be confounded by factors such as prescriber bias, socioeconomic status, access to private healthcare, or underlying clinical prognosis. The utilisation of nutritional supplements has frequently been observed to correlate with unmeasured health behaviours and social determinants, which in turn may influence the outcomes of treatment. Consequently, the relationships documented in this study should be regarded as exploratory, hypothesis-generating associations that require validation through prospective, multicentre studies incorporating ablation analysis, larger cohorts, and detailed nutritional and clinical data. “*

Reviewer 3 : 4- Figures and tables remain dense and sometimes unclear (especially Table 3). Typos, formatting errors, and awkward English phrasing persist in multiple places (e.g., “higest,” “phytolexin,” “vitamine c”). Method descriptions (e.g., ABC algorithm pseudocode) are overly technical for a biomedical journal and lack clarity on practical implications.

Respond : Thank you for this valuable suggestion. We have carefully revised the manuscript to correct grammatical errors. In addition, the paper has undergone thorough proofreading to ensure that it meets academic writing standards in terms of clarity, style, and consistency. These improvements enhance the overall readability and professionalism of the manuscript.

Reviewer 3 : 5- ABC parameters (20 bees, 10 iterations) remain inadequately justified. No sensitivity analysis was performed.
Findings are exploratory, yet sections of the discussion still suggest clinical implications. The study does not measure actual pregnancy/live birth outcomes—only embryo transfer success—limiting clinical utility.

Respond : Thank you for this valuable suggestion. we note the values and briefly say they were “chosen to balance computational efficiency and convergence, in line with prior studies reporting similar ranges” (and cite Karaboga & Basturk, 2007; Akay & Karaboga, 2012). And this explanation was added to discussion section.

“*These values were chosen to balance computational efficiency with convergence stability. However, no formal sensitivity analysis was conducted to evaluate the effect of parameter variation on model performance*”

It is imperative to emphasise the following point concerning the clinical implications. The following paragraph was incorporated into the discussion section with the objective of clarifying that the findings should be interpreted within the methodological context rather than the clinical context.

“ *Moreover, while the hybrid LR–ABC framework demonstrated improved predictive accuracy for embryo transfer outcomes, these findings should be regarded as exploratory and methodological rather than clinical. The study did not measure pregnancy or live birth rates, and therefore cannot inform treatment efficacy or reproductive prognosis. As such, the model’s scope is limited to predicting the likelihood of embryo transfer success within the observed dataset and does not extend to broader clinical outcomes.* ”

Reviewer 3 : 6- My recommendation for the author's improvement of the manuscript is to reframe the entire paper as a methodological proof-of-concept, not as clinical evidence of supplement efficacy. Explicitly remove any implication that omega-3 or folic acid are treatments; present them only as correlates.

Respond : Thank you for your valuable comments. All sentences pertaining to implications or clinical evidence are replaced by sentences demonstrating statistical associations with reproductive outcomes. The following replacement process is to be followed:

Orginal 1: These findings suggest a beneficial role of resveratrol supplementation on reproductive outcomes.

Revised 1: Previous studies have reported that resveratrol supplementation shows statistical associations with reproductive outcomes; however, these findings should be regarded as observational and not indicative of therapeutic efficacy.

Orginal 2: These findings suggest a beneficial role of resveratrol supplementation on reproductive outcomes.

Revised 2: Previous studies have reported that resveratrol supplementation shows statistical associations with reproductive outcomes; however, these findings should be regarded as observational and not indicative of therapeutic efficacy.

Orginal 3: These findings underscore the potential benefit of preconceptional vitamin B supplementation for enhancing IVF success.

Revised 3: Some studies have observed correlations between preconceptional vitamin B-complex use and IVF success indicators; however, such associations are exploratory and may be influenced by unmeasured confounding factors rather than reflecting a causal or therapeutic effect.

Orginal 4: Based on the highest F-score … the most effective for subfertility treatment are omega 3, folic acid, dietician support, phytoalexin, vitamin C, and vitamin B6.

Revised 4: Based on the highest F-score and LIME interpretability results, omega-3, folic acid, dietician support, phytoalexin, vitamin C, and vitamin B6 emerged as the most influential predictors associated with embryo-transfer outcomes.

Orginal 5: According to Fig. 6, omega-3 is the most effective active substance for subfertility treatment. The higher the omega-3 intake, the lower the risk of subfertility regardless of age

Revised 5: According to Fig. 6, omega-3 appeared as one of the most influential predictive variables in the model. Higher recorded omega-3 use was statistically associated with higher embryo-transfer success rates in this dataset, but this relationship should not be interpreted as causal.

Reviewer 3 : 7- There should be a stronger emphasis on overfitting risk and lack of generalizability. Stress that binary supplement coding is a major weakness.

Respond : Thank you for your valuable suggestion. We add the below paragraph for meeting the Overfitting risk and limited generalizability problem

“ *Although the hybrid LR–ABC models achieved relatively high predictive performance, these findings should be interpreted with caution given the limited sample size and the potential for overfitting. The application of five-fold cross-validation and SMOTE helped mitigate, but could not fully eliminate, this risk. Consequently, the observed performance metrics may reflect model behavior specific to the present dataset rather than a generalizable predictive pattern. Future studies using larger, independent, and multicenter datasets are required to validate the framework’s robustness.”*

and in the research limitation and future work section, paragraph starting with “Thirdly, only supplement intake was encoded as binary…” identifies binary supplement coding as a major weakness.

Reviewer 3 : 8- Improve validation by adding ablation or sensitivity analyses (e.g., performance of ABC vs. baseline LR without optimization). Consider external dataset testing (even partial) if available.

Respond_1 : Thank you for your valuable contribution. We added below description to the Results or Discussion section

“ *A sensitivity analysis was conducted by contrasting baseline Logistic Regression (LR) models with their ABC-optimized counterparts under the same cross-validation conditions in order to assess the autonomous contribution of the Artificial Bee Colony (ABC) optimizer. The hybrid ABC models continuously produced improved recall and 3–6% higher F1-scores, demonstrating that optimization, not chance, is the source of performance gains. The framework was tested using stratified resampling to mimic external generalization behavior even though external validation data were not available*.”

Simplify tables: keep full data in appendices, present concise summary tables in the main text. Improve figure quality and captions (ensure alignment with text). Proofread thoroughly for grammar, terminology consistency, and readability.

Respond_2 : Thank you for this valuable suggestion. We have carefully revised the manuscript to eliminate repetitions and correct grammatical errors. We simplyf the Table 1 remove the description column. We check the quality of the pictures (300 DPI). We add suitable caption to the Table 3-4 caption. In addition, the paper has undergone thorough proofreading to ensure that it meets academic writing standards in terms of clarity, style, and consistency. These improvements enhance the overall readability and professionalism of the manuscript.

Reviewer 3 : 9- Provide stronger justification for ABC parameter settings, citing prior literature in biomedical ML applications. Explain why ABC was chosen over other optimizers (e.g., GA, PSO) in the IVF context.

Respond : Thank you for this valuable contribution. We explain the reason of selecting of the ABC algoritm among the GA, PSO. We added below expression to the Proposed Models section. And added references to the reference section.

“ *The Artificial Bee Colony (ABC) algorithm was selected as the optimization component due to its proven efficiency and simplicity in continuous parameter tuning problems. ABC balances exploration and exploitation through its employed and onlooker bee phases, making it robust for small to moderate-sized biomedical datasets[**41**,* *42**]. Compared with alternative metaheuristics such as Genetic Algorithms (GA) and Particle Swarm Optimization (PSO), ABC offers fewer control parameters and faster convergence, making it a suitable choice for optimizing logistic regression coefficients in limited-sample biomedical prediction tasks such as IVF outcome modeling. ABC can achieve competitive or superior performance to GA and PSO while maintaining a simpler structure and requiring fewer hyperparameters [**43**,* *44**]. Subsequent studies have further highlighted ABC’s adaptability and search efficiency, emphasizing its robustness and minimal parameter tuning demands in continuous optimization problems [**45**]. These characteristics make ABC particularly appropriate for small, noisy, and heterogeneous biomedical datasets, where over-parameterization of the optimization process may lead to overfitting. “*

Reviewer 3 : 10- Highlight that results are associational and cannot inform treatment decisions. Suggest that future studies incorporate prospective data, nutrient dosages, and longitudinal outcomes.

Respond : Thank you for this valuable contribution. The primary focus of the present manuscript is associational thought, with a concomitant discussion of future research directions. And we added below expression to the research limitation and future work.

*“ The results of the study should be interpreted as associational rather than causative. While statistical patterns between nutritional supplement variables and IVF outcomes are identified by machine learning models, these associations do not necessarily indicate the effectiveness of treatment or clinical benefit. The present study adopts a methodological approach and does not seek to provide recommendations for personalised treatment or clinical decision-making. In order to support causal inference and validate these initial associations in larger and more diverse patient populations, future research should strive to include prospective data collection, accurate nutrient dosage information, and longitudinal follow-up outcomes. “*

Reviewer 3 : 11- The title and abstract, which are just a guide for the authors, should be written in the following manner.

The title and abstract, which are just a guide for the authors, should be written in the following manner:
Predicting IVF Outcomes Using a Logistic Regression–ABC Hybrid Model: A Proof-of-Concept Study on Supplement Associations
(This emphasizes prediction, methodology, and associations — not treatment or causality.)
Background
Machine learning models are increasingly applied to assisted reproductive technologies (ART), but most studies rely on conventional algorithms with limited optimization. This proof-of-concept study investigates whether a hybrid Logistic Regression–Artificial Bee Colony (LR–ABC) framework can improve predictive performance in in vitro fertilization (IVF) outcomes, while generating interpretable, hypothesis-driven associations with nutritional and pharmaceutical supplement use.
Methods
A retrospective dataset of 162 women undergoing IVF was analyzed. Clinical, demographic, and supplement variables were pre-processed into 21 predictors. Four algorithms (K-Nearest Neighbors, Classification and Regression Tree, Support Vector Machine, and Random Forest) were implemented alongside their LR–ABC hybrid counterparts. Model performance was evaluated using 5-fold cross-validation with SMOTE to address class imbalance. Local Interpretable Model-agnostic Explanations (LIME) were used to provide interpretability.
Results
Across all algorithm families, LR–ABC hybrids outperformed baseline models (e.g., Random Forest: 85.2% → 90.7% accuracy). LIME explanations identified omega-3, folic acid, and dietician support as influential features in individual predictions. However, given the small sample size, binary representation of supplements, and absence of external validation, the observed improvements and associations should be regarded as exploratory rather than definitive.
Conclusion
The LR–ABC hybrid model demonstrates methodological potential for enhancing prediction and interpretability in IVF research. Findings regarding supplement associations are hypothesis-generating and not clinically directive. Future studies with larger, multi-center datasets, including detailed dosage and dietary data, are needed to validate and extend this framework.
Keywords: Hybrid Machine Learning, IVF Prediction, Nutritional Supplements, Metaheuristic Optimization, Artificial Bee Colony
In this version, emphasis is on the methodological proof of concept.

Respond : Thank you for your valuable suggestion. We rewrite the abstract in this concept. We revised the keywords.
