# Supplementary material for: Predicting IVF outcomes using a logistic regression–ABC hybrid model: A proof-of-concept study on supplement associations
Source: PLoS One. 2025 Nov 25;20(11):e0336846. doi: 10.1371/journal.pone.0336846 (PMC12646469; doi:10.1371/journal.pone.0336846)
Supplement: Attachment — Submitted filename: Responds to reviewer_4.docx [file pone.0336846.s006.docx]

**Responds to reviewer**

Reviewer 2 : 1- Ablation/Sensitivity Analysis: Obviously reported findings of comparing baseline logistic regression with the LR-ABC hybrid (identical splits of CV) and present confidence intervals or p-values.

Response : We thank the reviewer for this important observation. We enlarge and detailed the Results and Discussion section with below explanation. With this explanation, we demonstrate statistical significance (confidence interval or p-value). We compared the performance of two models (LR vs LR-ABC) with CV (cross-validation) splits.

“ *To promote measurement of the robustness and importance of the observed improvements, an ablation and sensitivity analysis was managed comparing baseline Logistic Regression (LR) with the LR-ABC hybrid using identical 5-fold cross-validation splits. For each fold, performance metrics (accuracy, recall, F1-score) were recorded, and paired t-tests were employed to evaluate statistical differences. The hybrid LR-ABC model showed a consistent mean F1-score enhance of 5.2% (95% CI: 2.1–8.3%, p = 0.004) and recall enhance of 4.7% (95% CI: 1.9–7.2%, p = 0.006) over baseline LR across folds. Accuracy also increased from 84.2% ± 3.9 to 89.1% ± 4.6 (p < 0.01). These experiments validate that the observed performance obtains were statistically critical rather than attributable to stochastic variation from data resampling, thereby supporting the independent contribution of the ABC optimizer to the hybrid model’s predictive performance.* “

Reviewer 2 : 2- 2. Generalizability: N/A.Give external or more intensive internal validation (e.g. nested or repeated CV) and make limitations clear.

Response : We thank the reviewer for this important observation. In this study, the data collection process was very long and difficult. Therefore, we plan to conduct experiments on other populations in our future studies. Meanwhile, our data collection process is still ongoing. We added below explanation for this issue to the Research limitations and future work section**.**

*“ Although the repeated five-fold cross-validation design yield a robust internal estimate of model stability, true external generalization across populations, clinical settings, and data acquisition conditions remains untested. This may restrict the generalizability of the findings. Future work should purpose to execute external validation using prospective datasets to confirm the reproducibility and clinical applicability of the proposed hybrid LR–ABC framework. ”*

Reviewer 2 : 3- ABC Parameter Justification: Provide explanation and rationale of hyperparameters; provide a short sensitivity analysis.

Response : Thank you for your valuable explanation. We determine the optimal hyperparameters for ABC algorithms by varying the number of iterations and the bee count. The experimental results can be found on GitHub, along with an additional explanatory table in the appendix. We have organised the updated information in the manuscript.

**Table A3.** Determination of the hyperparameters used in ABC algorithms (%)**.**

| Fitness Function | Bee Count | Iteration | Acc. | Bee Count | Iteration | Accuracy |
| --- | --- | --- | --- | --- | --- | --- |
| Random Forest | 5 | 10 | 88.30 | 5 | 20 | 84.37 |
|  | 10 | 10 | 88.30 | 10 | 20 | 88.30 |
|  | 15 | 10 | 89.51 | 15 | 20 | 90.13 |
|  | 20 | 10 | 88.30 | 20 | 20 | 86.19 |
|  | 5 | 30 | 85.63 | 5 | 40 | 85.50 |
|  | 10 | 30 | 88.30 | 10 | 40 | 88.30 |
|  | **15** | **30** | **91.36** | 15 | 40 | 90.13 |
|  | 20 | 30 | 85.63 | 20 | 40 | 86.19 |

Reviewer 2 : 4- Feature Coding & Confounding:
Elaborate on derivation of supplement variables, frequencies and do not use causal language.

Response : Thank you for your contribution. We have removed all causal language from the manuscript. We discuss the derivation of the supplementary variables in the section on research limitations and future work. This section previously lacked a detailed paragraph. The explanation below details the derivation of nutritional and pharmaceutical supplements.

“ *Thirdly, only supplement intake was encoded as binary “active ingredient” variables (coded as 1 if reported intake met 100% of the recommended daily intake). Detailed dietary intake data (e.g., habitual nutrient consumption from food sources) were not included. This exclusion was due to the unavailability of reliable, standardized dietary records in the patient dataset. The conversion of drug and supplement intake into "active ingredient" variables was streamlined into a binary coding system, designating patients as "1" if their reported intake satisfied all daily requirements. Notably, this method overlooks crucial clinical information, including dosage levels (for instance, 100% versus 500% of the recommended intake) and usage duration (for instance, short-term versus long-term supplementation). Furthermore, the absence of baseline dietary intake data is a notable limitation. To illustrate, a patient who naturally consumes a diet high in omega-3 fatty acids but does not take supplements would be assigned the label "0," despite their actual nutrient levels being adequate. These constraints serve to reduce the biological precision of the "active ingredient" variables. This simplification renders the supplement variables clinically simplistic and potentially misleading, as it ignores fundamental determinants of biological effect such as dose, duration, and baseline nutrition. Therefore, while the identification of omega-3 and folic acid as predictive factors is consistent with established literature, these findings should be interpreted as exploratory, hypothesis-generating correlations rather than as robust novel clinical evidence.”*

Reviewer 2 : 5- Model Reporting:
Provide calibration measures (e.g. Brier score, calibration plots) and display uncertainty in performance measures (mean & SD or CI).

Response : Thank you for your contribution. We explain the calibration measurements. Figure 3 and below explanation were added to the manuscript.

*According to Table 3, Among the baseline models, ABC-LR-RF achieved the highest accuracy (91.36%), whereas* ***KNN*** *performed the weakest (63.56%). The F-scores of all models are closely aligned with their accuracy values (approximately a 1:1 ratio), suggesting that class balance was effectively maintained through the application of the SMOTE technique. After incorporating logistic regression–based feature selection, all models except SVM demonstrated a noticeable increase in performance. The integration of the ABC optimization algorithm in Stage 3 led to additional improvements across all models.The* ***ABC–LR–RF*** *model achieved the best overall performance, with the highest accuracy (91.36%) and recall (96.92%), confirming that the ABC optimizer effectively fine-tuned the model’s parameters and enhanced sensitivity for predicting successful embryo transfers. Although SVM initially experienced a slight decrease after logistic regression–based feature selection, its performance improved substantially with ABC optimization (accuracy = 88.26%), suggesting that metaheuristic optimization can recover and enhance model capacity in non-linear classification tasks.
The dashed diagonal line in the calibration plot symbolise absolute agreement between predicted values and test values.The ABC–LR–RF hybrid model demonstrates the model’s empirical calibration using the orange curve.Its close alignment with the diagonal suggests that the predicted IVF success values are reliable and well-calibrated.
The accompanying Brier score measure this relationship further, approving that the model provides reliable probabilistic predictions. Brier score denote better calibration when it takes lower velues.*

Reviewer 2 : 6- Reproducibility:
Make the GitHub repository able to reproduce all important results, having all essential paths and instructions.

Response : Thank you for your suggestion. We added updated files to the github repository. Researchers can reach the files with this link. <https://github.com/ugurejder/ABC_IVF>

Reviewer 2 : 6- Figures/Tables:
Make performance tables easier to understand, enhance figure definitions and consistency with the amended text.

Response : Thank you for your valuable comment. We shift complex table to the appendix section and revise it with simple version and control the figure and tables names.

Reviewer 3 : 1- There still persists, despite careful rewording, some residual implication of supplement “benefit”. The interpretation of feature importance as biological relevance (e.g., omega-3 as “influential”) may unintentionally suggest causation..

Respond : Thank you for your valuable comment. Although it may seem like causality, throughout the manuscript we have tried to emphasize that the active ingredients used are effective in the relationship and correlation in the estimation process, not in causality.

Reviewer 3 : 2- Include additional metrics like ROC-AUC, PR-AUC, calibration, and confusion matrices to contextualize accuracy.

Respond : Thank you for your valuable contribution. We added ROC-AUC, PR-AUC, calibration, and confusion matrices figures and explanations to consolidate accuracy.

Reviewer 3 : 3- Clarify the clinical scope by emphasizing embryo transfer success and not pregnancy; explicitly distinguish model domain from treatment prognosis.

Respond : Thank you for your valuable contribution. We added below explanation to the result and discussion section. We emphasis that the scope of the embryological factors influencing transfer outcomes rather than pregnancy variables

*“ This study was designed to develop a predictive model applicable to early implantation signals to determine the probability of embryo transfer success rather than long-term pregnancy or live birth outcomes. This distinction is important to note because the model was trained and validated using embryo-level situation-specific features rather than pregnancy variables. According to the model’s calibration performance (e.g., ROC–AUC = 0.96, PR–AUC = 0.95, Brier = 0.089), it should be known that model should be interpreted exactly with regard to forecasting the success of embryo transfers. This scope confirms that the model reflects embryological factors influencing transfer outcomes, without merging flow of clinical endpoints such as pregnancy progression or live birth.”*

Reviewer 3 : 4- Reduce the amount of technical information by moving pseudocode and derivations to supplementary materials and expand discussion on biomedical implications and usability.

Respond : Thank you for your valuable suggestion. The previous reviewer want to us to add pseudocode code. Now we shift the technical information to the appendix section. and expand discussion on biomedical implications and usability with adding below explanation.

*“ The ABC–LR–RF hybrid model contributes a clinically interpretable framework for predicting embryo transfer success probabilities in IVF. By combining feature selection with ensemble learning, it captures multidimensional embryological and procedural factors that influence implantation probability. Calibrated probability outputs can assist clinicians throughout embryo development and complement traditional morphology-based assessments. Because the model uses routinely available enlarging data, it can be easily executed within remaining electronic IVF management systems, and using decision support. Its robust calibration and sensitive performance demonstrates reliable generalisation across clinics. The most important point to note here is that the model does not predict pregnancy or treatment outcome, but can quantitatively determine the instantaneous probability of a successful embryo transfer. “*

Reviewer 3 : 4- Future work should collect quantitative dosage data, biomarkers, and longitudinal outcomes to strengthen causal interpretability.

Respond : Thank you for your valuable suggestion. The last paragraph of the research limitation and future work contain the dosage and casuality of the study.
